# Intrathecal daptomycin use in a challenging case of *Enterococcus faecium* ventriculitis

**DOI:** 10.1099/acmi.0.000230

**Published:** 2022-04-08

**Authors:** Ankush Dhariwal, Rachel Leff, Mike Allen, Benny Cherian

**Affiliations:** ^1^​ Barts Health NHS Trust, Royal London Hospital, Whitechapel Rd, London E1 1BB, UK; ^2^​ Merck Sharp & Dohme (UK) Limited, 120 Moorgate, London, EC2M 6UR, UK

**Keywords:** daptomycin, ventriculitis, *Enterococcus faecium*, VRE, intrathecal, external ventricular drain

## Abstract

Treatment of ventriculitis caused by enterococci can be challenging, and antibiotic options are limited. We describe a case of device-related ventriculitis caused by vancomycin-resistant *Enterococcus faecium *, refractory to initial antibiotics. Our management approach included intrathecal daptomycin. There were no attributable adverse events, and the patient remained infection-free following ventriculo-peritoneal shunt insertion and cessation of antibiotics.

## Introduction

Management of vancomycin-resistant enterococcal (VRE) device-associated ventriculitis is extremely challenging. This is for a number of reasons, including enterococcal biofilm formation, limited antibiotic options, pharmacokinetic considerations about central nervous system penetration, and potential toxicity of the few available agents. It is possible that bactericidal agents may be preferable to bacteriostatic agents, but bacteriostatic agents have been used successfully for this indication, and data on VRE ventriculitis management are limited [1]. Monitoring of patient response is also difficult, since typical markers of infection may not be present, and Glasgow coma score (GCS) may be reduced for a number of reasons in this patient group. Here, we describe a case in which we used intrathecal daptomycin alongside other agents.

## Case presentation

The patient was a male in his thirties with no past medical history who suffered a head injury following a fall from a height. He suffered subdural haematoma with mass effect, requiring a decompressive craniectomy and an external ventricular drain (EVD) insertion for raised intracranial pressure. He finished a course of piperacillin–tazobactam 4.5 g three times daily for ventilator-associated pneumonia on day 15 of admission, and continued to require an EVD for raised intracranial pressure.

On day 19 of admission, a cerebrospinal fluid (CSF) sample from the EVD grew *Enterococcus faecalis* (sensitive to amoxicillin and vancomycin). The patient’s microbiology results, antimicrobial therapy and surgical procedures are shown in Table S1 (available in the online version of this article). Intravenous (IV) vancomycin and intrathecal (IT) vancomycin was commenced, and IV vancomycin was later switched to amoxicillin 2 g three times daily. The EVD was therefore changed on day 20. However, a CSF sample from the new EVD on day 21 grew vancomycin-resistant *Enterococcus faecium* (VRE), with sensitivities as per Table 1.

**Table 1.** Sensitivities of VRE isolated from CSF

Antibiotic	Interpretation	Reported minimum inhibitory concentration (MIC; mg l^−1^)
Teicoplanin	R	>16
Vancomycin	R	>32
Tigecycline	S	≤0.25
Rifampicin	R	>2
Levofloxacin	R	>4
Imipenem	R	>8
Synercid	I	2
Linezolid	S	2
Chloramphenicol	S	≤8
Daptomycin	S*	4

*There is no EUCAST breakpoint for daptomycin for *E. faecium*, therefore the CLSI breakpoint of <4 was used as an ‘interpretive’ breakpoint.

Therefore, IV linezolid 600 mg twice daily was added on day 23, and IT vancomycin was stopped. CSF culture remained positive for VRE on day 25, and the EVD was removed. A new EVD was required on day 30 due to raised intracranial pressure, and a CSF sample was again positive for VRE, therefore IV chloramphenicol 1 g four times daily was added. Following this, cultures were negative on day 33, and the chloramphenicol was continued, with a view to inserting a ventriculo-peritoneal (VP) shunt following cure of infection, for long-term management of hydrocephalus.

On day 56, the patient developed bacteraemia with *Pseudomonas aeruginosa *. IV meropenem 1 g three times daily was commenced, and on day 57 the patient was diagnosed with worsening radiological appearances of ventriculitis, with new debris in the left temporal horn on CT head. Meropenem was increased to 2 g three times daily, and IV linezolid was recommenced for 3 days, as well as intrathecal (IT) gentamicin. The CSF white cell count was 2250 (70 % neutrophils, red cells 50), and culture was negative. By day 70, the CSF white cell count had fallen to 10 and it was culture-negative, and 16S ribosomal PCR was negative. Therefore, the patient was prepped for a VP shunt.

Given the previous VRE infections, and the risk of colonization of a VP shunt, we wished to provide some additional cover against VRE prior to shunt insertion. Our preference was to add in an agent with bactericidal activity against VRE, such as daptomycin, however given systemically, this has poor central nervous system penetration. We noted previous case reports of intrathecal daptomycin administration [1–4], with some positive results, and no significant toxicity.

Information about the drug, including stability, shelf life and filter compatibility, was gathered [5, 6], and a monograph was prepared. Since IT daptomycin is not licensed for this indication, appropriate local governance procedures were followed to approve this locally. Consent to administer intrathecal daptomycin was also sought from the patient’s next of kin. Intrathecal daptomycin 10 mg once daily was given via the EVD on days 70, 71 and 72, and a right-sided VP shunt was inserted on day 72. An intra-operative CSF showed a white cell count of 3, and was culture-negative. Due to the short-planned duration, no therapeutic drug monitoring of daptomycin was performed.

Creatine kinase was measured prior to starting IT daptomycin, and rechecked 2, 4 and 7 days after starting it. All values were within the normal range. A week after the first dose of daptomycin was given, full blood count, urea and electrolytes, liver function tests, calcium, phosphate and magnesium remained within normal range (except for stable anaemia, haemoglobin 105 g l^−1^, which was present prior to daptomycin administration.) No fever was noted post-daptomycin administration. The patient’s GCS remained stable, with no adverse events noted, and meropenem and chloramphenicol were stopped on day 76.

On day 78, 6 days after the last dose of daptomycin, the patient developed non-epileptiform focal movements of his right arm and leg, attributed by the neurology team to brain injury, with MRI evidence of damage to the left basal ganglia and corticospinal tract in the brainstem. A repeat lumbar puncture on day 80 showed no evidence of infection, with a CSF white cell count of 8, and culture and 16S PCR being negative. The patient’s GCS remained unchanged at around 10 throughout his admission, and unfortunately when last assessed 7 months after his admission, his level of awareness was formally diagnosed as being in a minimally conscious state, attributed to traumatic brain injury.

## Discussion

Published experience to date with intrathecal use of daptomycin remains sparse, and is limited to case studies. Intrathecal daptomycin may be a valuable option in managing such infections, considering the limited antibiotic options available, their toxicity and the fact that daptomycin is bactericidal against VRE. Variable dosage regimes have been reported for IT daptomycin, including 5 mg every 3 days, for 12 days [3], 5 mg daily for 7 days [4], 5 mg on alternate days for 7 weeks [1], and titration of dosing based on CSF drug levels, from 10 mg every 3 days, to 5 mg every 3 days, over 2 weeks [2]. In our case, intrathecal daptomycin was well tolerated, at a dose of 10 mg once daily for 3 days, with no adverse events attributable to its use despite direct central nervous system administration. In the context of a persistent VRE ventriculitis requiring multiple antibiotics, it may have also helped to prevent colonization of the VP shunt with VRE, thus ensuring sterility.

## Supplementary Data

Supplementary material 1
